# Bioactive Alkaloids from the Marine-Derived Fungus *Metarhizium* sp. P2100

**DOI:** 10.3390/jof8111218

**Published:** 2022-11-17

**Authors:** Guang-Shan Yao, Zhong-Lian Ma, Yao-Yao Zheng, Ling Lv, Jun-Qiu Mao, Chang-Yun Wang

**Affiliations:** 1 Fujian Key Laboratory on Conservation and Sustainable Utilization of Marine Biodiversity, School of Geography and Oceanography, Minjiang University, Fuzhou 350108, China; 2 Key Laboratory of Marine Drugs, The Ministry of Education of China, School of Medicine and Pharmacy, Ocean University of China, Qingdao 266003, China; 3 Laboratory for Marine Drugs and Bioproducts of Qingdao National Laboratory for Marine Science and Technology, Qingdao 266237, China; 4 Institute of Evolution & Marine Biodiversity, Ocean University of China, Qingdao 266003, China

**Keywords:** marine-derived fungus, *Metarhizium*, alkaloids, anti-*Vibrio* activity, anti-inflammatory activity

## Abstract

The *Metarhizium* fungal species are considered the prolific producers of bioactive secondary metabolites with a variety of chemical structures. In this study, the biosynthetic potential of marine-derived fungus *Metarhizium* sp. P2100 to produce bioactive alkaloids was explored by using the one strain many compounds (OSMAC) strategy. From the rice solid medium (mixed with glucose peptone and yeast broth (GPY)), wheat solid medium (mixed with Czapek) and GPY liquid medium, one rare *N*-butenone spiroquinazoline alkaloid, *N*-butenonelapatin A (**1**), together with nine known compounds (**2**–**10**), were isolated and identified. Their structures were elucidated by analysis of the comprehensive spectroscopic data, including 1D and 2D NMR and HRESIMS, and the absolute configuration of **1** was determined by a single-crystal X-ray crystallographic experiment. *N*-butenonelapatin A (**1**) represents the first example of *N*-butenone spiroquinazoline with a rare *α*, *β*-unsaturated ketone side chain in the family of spiroquinazoline alkaloids. Compound **4** displayed antibacterial activity against *Vibrio vulnificus* MCCC E1758 with a minimum inhibitory concentration (MIC) value of 6.25 µg/mL. Compound **7** exhibited antibacterial activities against three aquatic pathogenic bacteria, including *V. vulnificus* MCCC E1758, *V. rotiferianus* MCCC E385 and *V. campbellii* MCCC E333 with the MIC values of 12.5, 12.5 and 6.25 μg/mL, respectively. Compounds **3** and **6** demonstrated anti-inflammatory activity against NO production induced by lipopolysaccharide (LPS) with the IC_50_ values of 37.08 and 37.48 μM, respectively. In addition, compound **1** showed weak inhibitory activity against the proliferation of tumor cell lines A-375 and HCT 116. These findings further demonstrated that fungi of the *Metarhizium* species harbor great potentials in the synthesis of a variety of bioactive alkaloids.

## 1. Introduction

In the last decade, marine-derived fungi have attracted increasing attention due to the chemical diversity and pharmacological properties of their secondary metabolites [1,2,3,4,5]. Alkaloids, an important class of secondary metabolites derived from marine organisms, have demonstrated great promise in the search for novel molecules with valued pharmacological activities, including antiviral, antibacterial, cytotoxic, antioxidant, antifungal and anti-inflammatory [6]. For example, the diketopiperazine alkaloid Plinabulin (formerly named as NPI-2358), produced by the marine-derived fungus *Aspergillus* sp., is in the clinical phase 3 trial to treat non-small cell lung cancer in combination with docetaxel [7]. Auranomide B has a new scaffold of quinazolin-4-one substituted with a pyrrolidin-2-iminium moiety, discovered from the marine-derived fungus *Penicillium aurantiogriseum*, displaying anticancer activity against HEPG2 cells with an IC_50_ value of 0.097 µmol/mL [8]. Asperversiamides J, a linearly fused prenylated indole alkaloid, isolated from the marine-derived fungus *Aspergillus versicolor*, exhibits considerable inhibitory activity of iNOS with an IC_50_ value of 5.39 µM, showing potential for developing next-generation anti-inflammatory drugs [9]. Varioxepine A, a 3*H*-oxepine-containing diketopiperazine-type alkaloid isolated from the marine algal-derived fungus *Paecilomyces variotii*, exhibits inhibitory activity against the plant pathogenic fungus *Fusarium graminearum* with an MIC value of 4 μg/mL [10]. In our previous study, a series of diketopiperazine-type alkaloids were isolated from marine-derived fungi, including thiodiketopiperazines [11], indole-diketopiperazines [12] and prenylated indole alkaloids [13]. For example, spirotryprostatin G, cyclotryprostatins F and G, a class of indole-diketopiperazines were isolated from marine-derived fungus *P. brasilianum*, of which spirotryprostatin G exhibited cytotoxicity against the HL-60 cell line with IC_50_ values of 6.0 μM, whereas cyclotryprostatins F and G exhibited cytotoxicity against the MCF-7 cell line with IC_50_ values of 7.6 and 10.8 μM, respectively [12].

Fungi from the *Metarhizium* genus are among the most common entomopathogens, thus, providing important commercial strains of bioinsecticides [14,15]. In addition, they are prolific producers of secondary metabolites; these compounds include polyketides, nonribosomal peptides, alkaloids, terpenoids and their hybrids. For example, destruxins (DTXs) are well-known as one class of *Metarhizium* metabolites. DTXs are a class of six-residue depsipeptides in which five amino acid units and a hydroxy acid together form a 19-membered macrocyclic ring. A variety of important activities of DTXs were reported, such as antitumoral, cytotoxic, antiviral, insecticidal, immunosuppressant, phytotoxic and antiproliferative effects [16,17]. Two 24-membered macrolides, JBIR-19 and JBIR-20, were also isolated from *Metarhizium* sp. These compounds could induce a striking elongated morphology of *Saccharomyces cerevisiae* at concentrations of 3.1 μmol/L and 13 μmol/L [18]. The *Metarhizium-flavoviride*-derived diterpenoids Viridoxins A and B showed insecticidal activity against the Colorado potato beetle (*Leptinotarsa decemlineata*) in a leaf contamination assay with IC_50_s of 40 and 51 ppm for A and B, respectively [19]. However, there are only a few reports on *Metarhizium* fungi from the marine environment to date.

During our continuing research on bioactive marine natural products from marine microorganisms [20,21,22,23,24], a novel *Metarhizium* species P2100 was isolated from the seawater of the Yellow Sea, China. Taking into account the diversity in the genome and secondary metabolome, it was reasonably expected that the structurally unique and bioactive compounds would be more likely to be identified from this new strain than the known species or strains. Therefore, *Metarhizium* species P2100 was used to explore its secondary metabolite potential, using the one strain many compounds (OSMAC) strategy. It was demonstrated that alkaloids dominate its metabolite profile. Notably, a new spiroquinazoline alkaloid together with nine known alkaloids were isolated and identified. The antimicrobial, antitumor and anti-inflammatory activities of the isolated compounds were also evaluated. Herein, we report the isolation, structure elucidation and bioassay of these compounds.

## 2. Materials and Methods

### 2.1. General Experimental Procedures 

The gene sample was obtained by MiniAmp PCR (ABI, Alexandria, VA, USA). Optical rotations were acquired by a JASCO P-1020 digital polarimeter (Jasco Corp., Tokyo, Japan). The UV spectra were recorded on a HITACHI UH 5300 UV spectrophotometer (Hitachi, Tokyo, Japan). The ECD data were acquired on a Chirascan Circular Dichroism spectrometer (Applied Photophysics Ltd., Leatherhead, UK). The IR spectra were recorded on a Nicolet-Nexus-470 spectrometer (Thermo Electron Co., Madison, WI, USA) using KBr pellets. The NMR spectra were acquired by a JEOL JEM-ECP NMR spectrometer (600 MHz for ^1^H and 150 MHz for ^13^C, JEOL, Tokyo, Japan) and a Bruker AVANCE Ⅲ (400 MHz for ^1^H and 100 MHz for ^13^C, Bruker, Fällanden, Switzerland) using TMS as an internal standard. HRESIMS were measured on a Thermo MAT95XP high resolution mass spectrometer (Thermo Fisher Scientific, Bremen, Germany) and ESIMS spectra on a Thermo DSQ EImass spectrometer (Thermo Fisher Scientific, Bremen, Germany). Single-crystal X-ray crystallographic analysis was performed on a Bruker D8 venture X-ray single crystal diffractometer (Bruker, Karlsruhe, Germany). Samples were analyzed on a Hitachi L-2000 HPLC system coupled with a Hitachi L-2455 photodiode array detector and using a C18 column (Kromasil 250 × 4.6 mm, 5 µm). The semi-preparative HPLC was conducted by a semi-preparative C18 column (Kromasil 250 × 10 mm, 5 µm). Silica gel (Qing Dao Hai Yang Chemical Group Co., Poli Town, China; 300−400 mesh), and the Sephadex LH-20 (Amersham Biosciences, Amersham, UK) was used for column chromatography (CC). Precoated silica gel plates (Yan Tai Zi Fu Chemical Group Co., China; G60, F-254) were used for thin-layer chromatography.

### 2.2. Isolation and Species Identification of Marine Fungus

The fungal strain P2100 was collected from seawater collected from the Qingdao Huiquan Bay, Yellow Sea, in August 2019. The fungus was identified as *Metarhizium* sp. fungus based on its morphological features and the sequence analysis of the ITS region (GenBank accession number: OP028052) of the rRNA gene and its derived phylogenetic analysis. This fungal strain was deposited in the Key Laboratory of Marine Drugs, Ministry of Education of China, School of Medicine and Pharmacy, Ocean University of China, Qingdao, China.

The genomic DNA of P2100 was isolated by using lysis buffer for microorganism to direct PCR (TaKaRa, Japan). The internally transcribed spacer (ITS) sequence was amplified from the genome DNA via PCR with primers (ITS1: 5′TCCGTAGGTGAACCTGCGG3′ and ITS4: 5′TCCTCCGCTTATTGATATGC3′) and then sequenced on a 3730xl DNA Analyzer (Applied Biosystems, San Francisco, CA, USA). ITS sequences (see Supplementary Materials) were then uploaded to the National Center of Biotechnology Information (NCBI) for BLAST analysis. MEGA.11 was used to construct the phylogenetic tree for ITS sequences of P2100 and its relatives based on the maximum likelihood method [25].

### 2.3. Fungal Fermentation and Metabolite Profile Analysis

The fungal conidia were inoculated in the sterilized liquid medium, cultured at 28℃, 150 rpm for 15 days. Eleven media used for fungal fermentation are listed in Table S4. For scaled-up fermentation, three media were used, including rice medium, wheat medium and GPY medium (Table S5). For HPLC analysis, the mobile system was phase A—water and B—methanol. Gradient elution was used: 0 min 5% B, 5 min 5% B, 10 min 30% B, 20 min 60% B, 30 min 100% B, 35 min 100% B, 40 min 5% B at a flow rate of 2 mL/min. The UV detection wavelength was at 210 nm.

### 2.4. Extraction and Isolation

The fungus *Metarhizium* sp. P2100 was cultured with the rice solid medium (mixed with GPY), wheat solid medium (mixed with Czapek) and GPY liquid medium, respectively. The solid fermentation mycelia were soaked in equal amounts of ethyl acetate (EtOAc) three times, respectively. The liquid fermentation broth and mycelia were separated through cheesecloth and extracted repeatedly with equal amounts of EtOAc three times, respectively.

The wheat mixed Czapek medium extracts were combined and evaporated in vacuo to afford an EtOAc extract (43.5 g). The EtOAc extract was isolated on silica gel column chromatography (CC) using a step gradient elution with petroleum ether/ethyl acetate (10:0 to 0:10, *v*/*v*) and ethyl acetate/methanol (10:0 to 0:10) to provide ten fractions (Fr.1−Fr.10). Fr.5 was separated by octadecyl silyl (ODS) reverse-phase silica gel CC using a step gradient elution with methanol/H_2_O (1:1 to 1:0, *v*/*v*) to provide five fractions (Fr.5.1−Fr.5.5). Fr.5.1 was separated by the semi-preparative high performance liquid chromatography (HPLC) eluted with methanol/H_2_O (45:55, *v*/*v*) to give compound **3** (6.6 mg). Fr.5.4 was separated by silica gel CC eluted with petroleum ether/ethyl acetate (3:1, *v*/*v*) and purified by the semi-preparative HPLC eluted with methanol/H_2_O (7:3, *v*/*v*) to give compound **4** (3.4 mg). Fr.6 was separated by silica gel CC using a step gradient elution with petroleum ether/ethyl acetate (4:1 to 0:1, *v*/*v*) to provide five fractions (Fr.6.1−Fr.6.5). Fr.6.3 was separated by silica gel CC eluted with petroleum ether/ethyl acetate (3:2, *v*/*v*) and purified with the semi-preparative HPLC eluted with methanol/H_2_O (1:1, *v*/*v*) to give compound **5** (14.1 mg). Fr.7 was separated by silica gel CC using a step gradient elution with petroleum ether/ethyl acetate (1:1 to 0:1, *v*/*v*) and ethyl acetate/methanol (1:1 to 0:1) to provide five fractions (Fr.7.1−Fr.7.5). Fr.7.3 was separated by Sephadex LH-20 CC eluted with dichloromethane/methanol (1:1, *v*/*v*) to give white precipitate; then, the precipitate was washed alternately using dichloromethane and methanol to give compound **2** (105.4 mg). Fr.7.5 was separated by semi-preparative HPLC with methanol/H_2_O (2:3, *v*/*v*) to yield compound **1** (8.0 mg).

The rice mixed GPY medium extracts were combined and evaporated in vacuo to afford an EtOAc extract (48.08 g). The EtOAc extract was isolated on silica gel CC using a step gradient elution with petroleum ether/ethyl acetate (10:0 to 0:10, *v*/*v*) and ethyl acetate/methanol (10:0 to 0:10) to provide eight fractions (Fr.1−Fr.8). Fr.1 was separated by ODS reverse-phase silica gel CC using a step gradient elution with methanol/H_2_O (3:7 to 1:0, v/v) to provide nine fractions (Fr.1.1−Fr.1.9), Fr.1.6 was separated by Sephadex LH-20 CC eluted with CH_2_Cl_2_/MeOH (1:1, *v*/*v*) to offer compound **6** (9.9 mg). Fr.3 was separated by ODS reverse-phase silica gel CC using a step gradient elution with methanol/H_2_O (2:3 to 1:0, *v*/*v*) to provide eleven fractions (Fr.3.1−Fr.3.11). Fr.3.3 was separated by Sephadex LH-20 CC eluted with dichloromethane/methanol (1:1, *v*/*v*) to give Fr.3.3.4; then, the white solid was washed with methanol to obtain compound **7** (5.7 mg). Fr.3.4 was separated by silica gel CC eluted with dichloromethane/methanol (40:1, *v*/*v*) and purified by the semi-preparative HPLC eluted with methanol/H_2_O (7:3, *v*/*v*) to give compound **8** (1.5 mg).

The GPY medium extracts were combined and evaporated in vacuo to afford an EtOAc extract (1.5 g). The EtOAc extract was isolated on silica gel CC using a step gradient elution with petroleum ether/ethyl acetate (10:0 to 0:10, *v*/*v*) and ethyl acetate/methanol (10:0 to 0:10) to provide six fractions (Fr.1−Fr.6). Fr.5 was separated by silica gel CC eluted with petroleum ether/ethyl acetate (1:3, *v*/*v*) and the ODS reverse-phase silica gel CC eluted with methanol/H_2_O (2:3 to 1:0, *v*/*v*) to give compounds **9** (2.5 mg) and **10** (3.2 mg).

### 2.5. Bioassay

The antibacterial activity was evaluated following the standards recommended by Yang [26]. three marine-derived pathogenic bacterial strains, *V. vulnificus* MCCC E1758, *V. rotiferianus* MCCC E385 and *V. campbellii* MCCC E333, were used, and ampicillin sodium was tested as a positive control.

The antifungal bioassay was conducted following the standards recommended by Yang [26]. Three pathogenic fungal strains, *Candida albicans* ATCC 24433, *C. tropicalis* ATCC 20962 and *C. parapsilosis* ATCC 22019, were tested, and amphotericin B was used as a positive control.

The 1,1-diphenyl-2-picryl-hydazyl (DPPH) scavenging assay was performed using the method described by Aquino [27]. The reaction mixture consisted of freshly prepared DPPH in ethanol (100 µmol/L) mixed with different concentrations of the tested compound. The reaction mixture was incubated for 20 min at room temperature in the dark, and the absorbance was recorded at 517 nm.

The Fe^3+^ reduction assay was performed using the method described by Aktumsek [28]. The reaction mixture consisted of freshly prepared 2,4,6-tripyridin-2-yl-1,3,5-triazine (TPTZ) in ultrapure water (100 µmol/L) mixed with different concentrations of the tested compound. The reaction mixture was incubated for 20 min at room temperature in the dark, and the absorbance was recorded at 593 nm.

The bioassay for NO production inhibitory activity was conducted as described by Xia [29]. The mouse macrophages were seeded in 96-well plates. In each well, lipopolysaccharide (LPS) (1 μg/mL) was added after treatment with or without the tested compound for 24 h. The NO production in the supernatant was detected by the Griess reaction. The absorbance at 540 nm was measured with a microplate reader. The NO concentration and the inhibitory rate were calculated through a calibration curve. Dexamethasone was used as the positive control. Experiments were operated in triplicate, and the data were reported as mean ± SD of three independent experiments; then, experiments with different concentrations of the tested compound were used to obtain the IC_50_ value.

The cytotoxic activity against human cancer cell lines was evaluated following the CCK-8 assay [30]. Twenty cell lines were used, including A549, MCF7, MKN-45, HCT 116, HepG2, HeLa, K-562, SF126, PA-1, 786-O, TE-1, 5637, DU 145, CAL-62, PATU8988T, HOS, A-375, A-673, FaDu and GBC-SD. Adriamycin was used as a positive control.

## 3. Results

### 3.1. Strain Isolation and Species Identification

**Figure 1:**
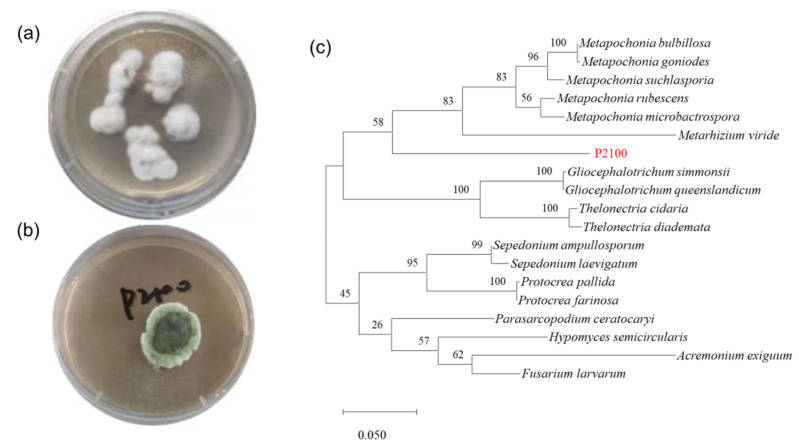
Species identification of the marine-derived fungal strain P2100. (**a**) Colony and (**b**) conidiation morphology of P2100 was observed by growth on a PDA plate for 3 and 7 days, respectively. (**c**) Phylogenetic tree of fungal strain P2100 based on ITS sequences by using maximum likelihood method with 1000 bootstraps.

*Metarhizium* sp. P2100 was isolated from seawater collected from the Yellow Sea, China, by the spreading plate technique and primarily identified as the *Metarhizium* genus by combining morphology and the alignment of ITS sequences (Figure 1). A phylogenetic tree was established based on the ITS sequence, which showed that P2100 diverged evolutionarily from known strain relatives and formed a relatively independent branch. Combined with the above analysis, P2100 was identified as a new species of *Metarhizium* from the marine environment.

### 3.2. HPLC analysis of Secondary Metabolite Profile of P2100 Cultured in Multiple Media

**Figure 2:**
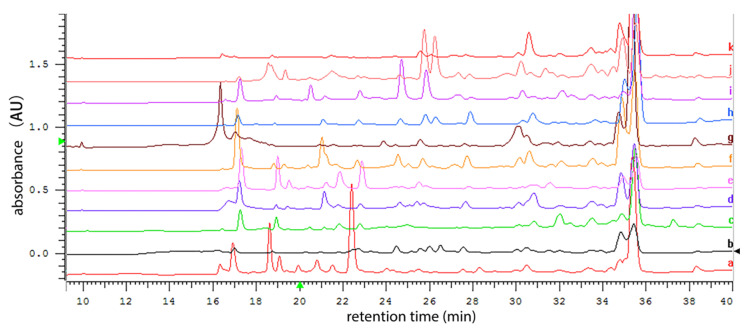
HPLC analysis of the secondary metabolite profile of P2100 cultured in 11 different media. a. YES medium; b. MEB medium; c. CYA medium; d. Czapek +1% tryptone medium; e. GPY medium; f. Czapek +1% yeast extract medium; g. PDB medium; h. Czapek medium; i. Starch medium; j. GMM medium; k. TBI medium.

So far, little is known about how to induce the production of secondary metabolites in marine-derived *Metarhizium* fungi. In this study, the one strain many compounds (OSMAC) strategy was adopted to induce the expression of secondary metabolite genes from *Metarhizium* sp. P2100. Eleven liquid media were used in fermentation to compare the P2100 metabolite profiles in different media. Among these media, Czapek medium was used as a chemically complete medium, YES medium had the highest C/N ratio, and conversely, GPY medium contained the lowest C/N ratio, while PDB and MEB media contained complex organic compounds. The HPLC profile analysis showed that the P2100 cultured in Czapek and GPY media exhibited higher chemical diversity than in other media (Figure 2). Considering that the filamentous fungi are commonly more prolific in rice solid state fermentation than those in the sole liquid-submerged fermentation, *Metarhizium* sp. P2100 was further cultured in solid medium (rice mixed with GPY and wheat mixed with Czapek) for scale-up fermentation.

### 3.3. Elucidation of Chemical Structures 

**Figure 3:**
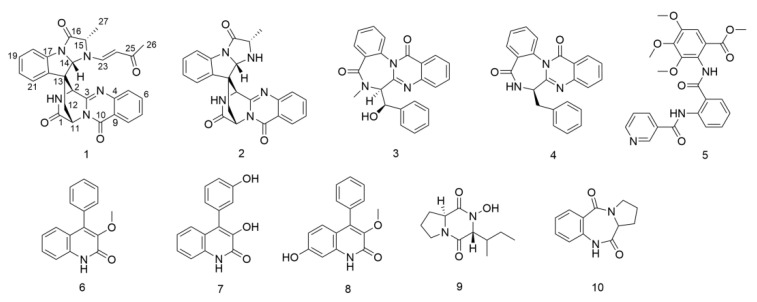
The structure of compounds 1–10.

The fungal strain *Metarhizium* sp. P2100 was cultured on rice solid medium (mixed with GPY), wheat solid medium (mixed with Czapek) and GPY liquid medium for scale-up fermentation, respectively. From the ethyl acetate extracts of the fermented products, ten alkaloids (**1–10**) were isolated using a combination of column chromatography including C18, silica gel, Sephadex LH-20 and semi-preparative HPLC. Compound (**1**) was identified as an unusual spiroquinazoline with a rare butenone side chain in alanine based on a detailed interpretation of its NMR and mass spectroscopic data, as well as single crystal diffraction analysis. The other nine known related alkaloids were assigned structurally by comparing their NMR, MS and OR data with those previously reported in the literature, including lapatin A (**2**) [31], benzomalvin E (**3**) [32], novobenzomalvin A (**4**) [33], methyl 3,4,5-trimethoxy-2-(2-(nicotinamido)benzamido)benzoate (**5**) [34], 3-*O*-methylviridicatin (**6**) [35], 3-hydroxy-4-(3-hydroxyphenyl)-2(1H)-quinolinone (**7**) [36], 7-hydroxy-3-methoxyviridicatin (**8**) [37], callyspongidipeptide A (**9**) [38] and cycloanthranilylproline (**10**) [39] (Figure 3). The structure of compound **6** was undoubtedly confirmed by its crystal data for the first time.

**Figure 4:**
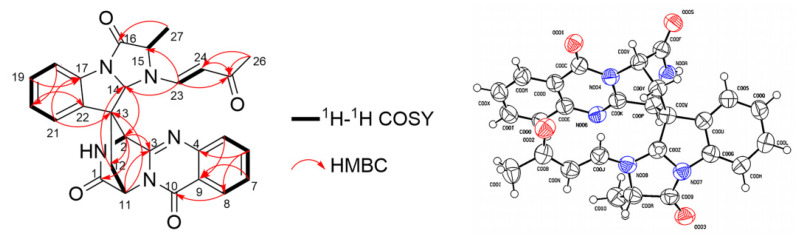
The key ^1^H–^1^H COSY and HMBC correlations of compound **1** (**left**) and the X-ray crystallographic structure of **1** (red circle: oxygen atom; blue circle: nitrogen atom) (**right**).

Compound **1** was isolated as a yellow amorphous powder. Its molecular formula was determined as C_27_H_23_O_4_N_5_ based on the protonated molecule peak at *m*/*z* 482.1810 [M + H]^+^ in HRESIMS, which required nineteen degrees of unsaturation. The UV spectrum of **1** exhibited the absorption peaks characteristic for a quinazoline ring (UV_max_ 216 nm). Careful analysis of the ^1^H and ^13^C NMR spectroscopic data (Table 1) revealed that compound **1** possessed similar structural features to those of lapatin A (**2**), a spiroquinazoline alkaloid derived from *P. lapatayae* [31]. Comparison of spectroscopic data for compounds **1** and **2** showed that one of the NH protons at *δ*_H_ 3.35 (d, *J* = 6.9 Hz, 1H) in the ^1^H NMR spectrum of **2** disappeared in that of **1**. Instead, proton signals resonating at *δ*_H_ 6.74 (d, *J* = 13.9 Hz, H-23), *δ*_H_ 4.74 (d, *J* = 13.9 Hz, H-24), *δ*_H_ 1.27 (s, H_3_-26) and carbon signals at *δ*_C_ 146.24 (C-23), *δ*_C_ 103.41 (C-24), *δ*_C_ 193.39 (C-25) and *δ*_C_ 25.31 (C-26) were observed in the NMR spectra of **1**. The HMBC correlations of the methyl protons H-26 to C-25, C-24 and H-23 to C-25 combined with the ^1^H-^1^H COSY correlation between H-23 and H-24 (Figure 4), suggested the presence of an *α*, *β*—unsaturated ketone side chain (−CH=CH−CO−CH_3_), which was consistent with the difference in the molecular formula. The HMBC correlations from H-23 to C-14, C-15 confirmed the linkage of the side chain to the N-atom in alanine. The *E* geometry of the double bonds was determined by the large trans coupling constants (*J =* 13.9 Hz). Therefore, the planar structure of compound **1** was determined as shown in Figure 3.

The relative configuration of **1** was determined on the basis of its NOESY spectrum. The NOESY correlation between H-12 (*δ*_H_ 3.02) and H-14 (*δ*_H_ 5.91) indicated the configuration at the center of the spiro C-13, and the correlation between H-27 (*δ*_H_ 1.70) and H-2 (*δ*_H_ 4.27) confirmed the inference mentioned above. The ECD spectrum of **1** (Figure S9) revealed the positive Cotton effects at 196, 234 and 274 nm and negative Cotton effects at 216, 245 and 297 nm, which was similar to the ECD data of lapatin A (**2**) [31]. It was speculated that the absolute configurations of **1** and **2** may be the same. Finally, a single X-ray diffraction analysis of **1** verified its plane structure and confirmed its absolute configuration. The final refinement of the Cu *Kα* data resulted in a 0.06(12) Flack parameter, which allowed an unambiguous assignment of the absolute configuration of **1** as 2*S*, 11*S*, 13*S*, 14*S*, 15*S* (Figure 4). Thus, compound **1** was determined as a butenone substitute of laptain A (**2**) at position N atom and named *N*-butenonelapatin A.

*N*-butenonelapatin A (**1**) represents the first reported *N*-butenone spiroquinazoline, belonging to the eleventh member of the spiroquinazoline alkaloid family. Intriguingly, comparison with those reported spiroquinazolines, *N*-butenonelapatin A (**1**) is a spiroquinazoline that is characterized by a rare *N*-butenone harboring at the *L*-alanine moiety instead of an *L*-alanine in lapatin A from *P. lapatayae* [31] and *N*-formyllapatin from *P. adametzioides* [40].

### 3.4. Spectroscopic and Spectrometric Data

*N*-butenonelapatin A(**1**): yellow powder; ${\left[\alpha \right]}_{D}^{25}$–55.8 (*c* 1.0, MeOH); UV (MeOH) λ_max_ (log *ε*) 216.5 (4.18), 225 (4.14) nm, 283 (3.86); CD (MeOH) λ_max_ (Δε) 196 (18.14), 216 (−17.97), 234 (11.25), 245 (−6.44), 274 (17.70), 297 (−19.40); IR (KBr) *ν*_max_ 3280, 2925, 1711, 1607, 1383, 1027 cm^–1^; ^1^H and ^13^C NMR (methanol-*d*_4_) see Table 1; HREMIMS *m*/*z* 482.1810 [M + H] ^+^, molecular formula: C_27_H_23_O_4_N_5_.

Crystal date for compound **1**: C_27_H_23_O_4_N_5_ *Mr* = 481.50, Identification code: cu_1123_7_0m, Temperature: 150.0 K, Crystal system: monoclinic, Space group: P21, *a* =8.3725(2) Å, *b* = 12.6206(3) Å, *c* = 11.0040(3) Å, *α* = 90°, *β* = 101.146(2)°, *γ* = 90°, Volume 1140.82(5) Å^3^, Z = 2, *ρ*_calcg_/cm^3^ 1.402, *μ* = 0.793 mm^–1^, F(000) = 504.0, Crystal size = 0.2 × 0.15 × 0.1 mm^3^, Radiation Cu K*α* (*λ* = 1.54178), Reflections collected: 7782, Independent reflections: 4073 (*R*_int_ = 0.0258, *R*_sigma_ = 0.0417), Data/restraints/parameters: 4073/1/327, Goodness-of-fit on F^2^ = 1.107, Final *R* indexes (*I* ≥ 2*σ* (*I*)) *R*_1_ = 0.0612, *wR*_2_ = 0.1439, Final *R* indexes (all data) *R*_1_ = 0.0628, *wR*_2_ = 0.1461, Largest different peak/hole = 0.38/–0.23 e Å^−3^, Flack parameter = 0.06(12).

3-*O*-methylviridicatin (**6**): white crystal, ESI-MS *m*/*z*: 252.71 [M + H]^+^, molecular formula: C_16_H_13_O_2_N. ^1^H NMR (400 MHz, DMSO-*d*_6_) *δ*_H_ 12.07 (s, 1H, NH), 7.51 – 7.47 (m, 2H, H-3′,5′), 7.45 – 7.42 (m, 1H, H-4′), 7.38 (ddd, *J* = 8.3, 7.0, 1.4 Hz, 1H, H-7), 7.33 (dd, *J* = 8.3, 1.2 Hz, 1H, H-8), 7.30 – 7.27 (m, 2H, H-2′,6′), 7.05 (ddd, *J* = 8.1, 7.0, 1.2 Hz, 1H, H-6), 6.94 (dd, *J* = 8.1, 1.4 Hz, 1H, H-5), 3.65 (s, 3H, H-OCH_3_); ^13^C NMR (100 MHz, DMSO) *δ*_C_ 158.49 (C-2), 145.08 (C-3), 137.42 (C-4), 135.78 (C-8a), 133.50 (C-1′), 129.16 (C-2′, 6′), 128.57 (C-7), 128.42 (C-3′, 5′), 128.04 (C-4′), 125.72 (C-5), 121.97 (C-6), 119.86 (C-4a), 115.11 (C-8), 59.44 (3-OCH_3_).

Crystal date for **6**: C_16_H_13_O_2_N *Mr* = 251.27, Identification code: exp_12273, Temperature: 293(2) K, Crystal system: monoclinic, Space group: C2/c, *a* =17.9000(6) Å, *b* = 15.7731(6) Å, *c* = 9.2998(4) Å, *α* = 90°, *β* = 99.430(4)°, *γ* = 90°, Volume 2590.20(17) Å^3^, Z = 8, *ρ*_calcg_/cm^3^ 1.289, *μ* = 0.688 mm^−1^, F(000) = 1056.0, Crystal size = 0.120 × 0.120 × 0.110 mm^3^, Radiation Cu K*α* (*λ* = 1.54184), Reflections collected: 4337, Independent reflections: 2316 (*R*_int_ = 0.0207, *R*_sigma_ = 0.0261), Data/restraints/parameters: 2316/0/174, Goodness-of-fit on F^2^ = 1.047, Final *R* indexes (*I* ≥ 2*σ* (*I*)) *R*_1_ = 0.0405, *wR*_2_ = 0.1087, Final *R* indexes (all data) *R*_1_ = 0.0498, *wR*_2_ = 0.1164, Largest different peak/hole = 0.19/–0.14 e Å^−3^. 

### 3.5. Bioassays

All of the isolated compounds were tested for their antibacterial, anti-fungal, anti-inflammatory, DPPH scavenging activity and Fe^3+^ reduction ability, and compound **1** was tested for its antitumor activity. It was found that compound **4** displayed antibacterial activity against *Vibrio vulnificus* MCCC E1758 with a MIC value of 6.25 µg/mL, and compound **7** exhibited antibacterial activities against *V. vulnificus* MCCC E1758, *V. rotiferianus* MCCC E385 and *V. campbellii* MCCC E333 with MIC values of 12.5 μg/mL, 12.5 μg/mL and 6.25 μg/mL, respectively. (Table S1). Compounds **3** and **6** showed moderate anti-inflammatory activities against NO production induced by LPS with IC_50_ values of 37.08 and 37.48 μmol/L, respectively (Table S2). Compound **1** was screened for inhibitory activity in 20 human tumor cell lines at a concentration of 10 μmol/L, demonstrating weak proliferation inhibitory activity against human melanoma cell A-375 and human colon cancer cell HCT 116 (Table S3). All of the isolated compounds showed no anti-fungal activity, no DPPH scavenging activity and no Fe^3+^ reduction ability.

## 4. Conclusions

In summary, the chemical diversity and biosynthetic potential of the marine-derived fungus *Metarhizium* sp. P2100 were explored by using the OSMAC strategy. From the culture of this strain with the solid medium (rice mixed with GPY and wheat mixed with Czapek, respectively) and GPY liquid medium, ten alkaloids were obtained and identified, including a new spiroquinazoline alkaloid, *N*-butenonelapatin (**1**), together with nine known analogs (**2**–**10**). *N*-butenonelapatin A (**1**) represents the first example of *N*-butenone spiroquinazoline with a rare *α*, *β—*unsaturated ketone side chain in the family of spiroquinazoline alkaloids. Compounds **4** and **7** exhibited antibacterial activities against *Vibrio* pathogenic bacteria. Compounds **3** and **6** demonstrated inhibitory activity against LPS induced NO production. Compound **1** displayed weak proliferation inhibitory activity against human tumor cell lines A-375 and HCT 116. In conclusion, the potential of the marine-derived fungus *Metarhizium* sp. P2100 to produce novel bioactive secondary metabolites is worthy of further exploration.

## Figures and Tables

**Table 1:** ^1^ H (400 MHz), ^13^C (100 MHz) NMR Data for Compound **1**.

Position	*δ*_C_ Type	*δ*_H_ Mult (*J* in Hz)	^1^H-^1^H COSY	HMBC
1	169.76			
2	55.83	4.27 (d, *J* = 4.0 Hz, 1H)	NH	C-1, 3, 12, 13
3	151.22			
4	147.60			
5	127.12	7.57 (d, *J* = 8.2 Hz, 1H)	H-6	C-7, 9
6	134.73	7.80 (ddd, *J* = 8.2, 7.1, 1.5 Hz, 1H)	H-5	C-4, 8
7	127.12	7.52 (m, 1H)	H-8	C-5, 6, 9
8	126.35	8.09 (dd, *J* = 8.2, 1.5 Hz, 1H)	H-7	C-4, 6, 10
9	119.86			
10	158.09			
11	52.87	5.58 (t, *J* = 2.9 Hz, 1H)	H-12	C-1, 3, 13
12	35.75	3.02 (dd, *J* = 15.3, 3.4 Hz, 1H), 2.75 (dd, *J* = 15.3, 2.4 Hz, 1H)	H-11	C-11, 13, 14
13	51.79			
14	85.36	5.91 (s, 1H)		C-2, 12, 16, 13, 23
15	61.41	4.43 (q, *J* = 6.9 Hz, 1H)	H-27	C-16, 23, 27
16	169.08			
17	136.38			
18	115.87	7.51 (m, 1H)		C-20, 22
19	129.56	7.49(m, 1H)	H-20	C-17, 21
20	126.07	7.35 (td, *J* = 7.5, 1.6 Hz, 1H)	H-19,21	C-18, 22
21	126.16	7.19 (d, *J* = 7.5 Hz, 1H)	H-20	C-13, 17, 19
22	136.99			
23	146.24	6.74 (d, *J* = 13.9 Hz, 1H)	H-24	C-14, 15, 25
24	103.41	4.74 (d, *J* = 13.9 Hz, 1H)	H-23	C-26
25	193.39			
26	25.31	1.27 (s, 3H)		C-25
27	16.97	1.70 (d, *J* = 6.9 Hz, 3H)	H-15	C-16
NH		9.26 (d, *J* = 4.0 Hz, 1H)	H-2	

## Data Availability

Not applicable.

## Supplementary Materials

The following supporting information can be downloaded at: www.mdpi.com/article/10.3390/jof8111218/s1. Figures S1–S9: ^1^H NMR, ^13^C NMR, HSQC, ^1^H-^1^H COSY, HMBC, HRESIMS, IR and CD spectra of compound **1** Tables S1–S3: The activity results about antibacterial, anti-inflammatory and anti-tumor and the rDNA-ITS sequence of the fungal strain *Metarhizium* sp. P2100
