# Supplementary material for: Bioactive Alkaloids from the Marine-Derived Fungus Metarhizium sp. P2100
Source: J Fungi (Basel). 2022 Nov 17;8(11):1218. doi: 10.3390/jof8111218 (PMC9698479; doi:10.3390/jof8111218)
Supplement: Supplementary file 1 [file jof-08-01218-s001.zip › jof-2006332-supplementary.pdf]

Bioactive alkaloids from the marine-derived fungus *Metarhizium* sp. P2100

Guang-Shan[1#] Yao, Zhong-Lian Ma[2,3,4#], Yao-Yao Zheng[2,3,4#], Ling Lv[2,3,4], Jun-Qiu Mao[2,3,4], Chang-Yun Wang[2,3,4*]

[1] Fujian Key Laboratory on Conservation and Sustainable Utilization of Marine Biodiversity, Institute of Oceanography, Minjiang University, Fuzhou 350108, China
[2] Key Laboratory of Marine Drugs, the Ministry of Education of China, School of Medicine and Pharmacy, Ocean University of China, Qingdao 266003, China
[3] Laboratory for Marine Drugs and Bioproducts, Qingdao National Laboratory for Marine Science and Technology, Qingdao 266237, China
[4] Institute of Evolution & Marine Biodiversity, Ocean University of China, Qingdao 266003, China
* Correspondence: changyun@ouc.edu.cn (C.-Y.W.); Tel.: +86-0532-8203-1536 (C.-Y.W.)
\# These three authors contributed equally to this work

List of Supplementary Material

Figure S1. ^{1}H NMR (400 MHz, DMSO-d_6) spectrum of **1**

Figure S2. ^{13}C NMR (100 MHz, DMSO-d_6) spectrum of **1**

Figure S3. HSQC (DMSO-d_6) spectrum of **1**

Figure S4. ^{1}H-^{1}H COSY (DMSO-d_6) spectrum of **1**

Figure S5. HMBC (DMSO-d_6) spectrum of **1**

Figure S6. NOESY (DMSO-d_6) spectrum of **1**

Figure S7. HRESIMS spectrum of **1**

Figure S8. IR spectrum of **1**

Figure S9. CD spectrum of **1**

Table S1. The antibacterial activity results (only sections are shown)

Table S2. The anti-inflammatory activity results (only sections are shown)

Table S3. The anti-tumor activity results (only sections are shown)

The rDNA-ITS sequence of the fungal strain *Metarhizium* sp. P2100

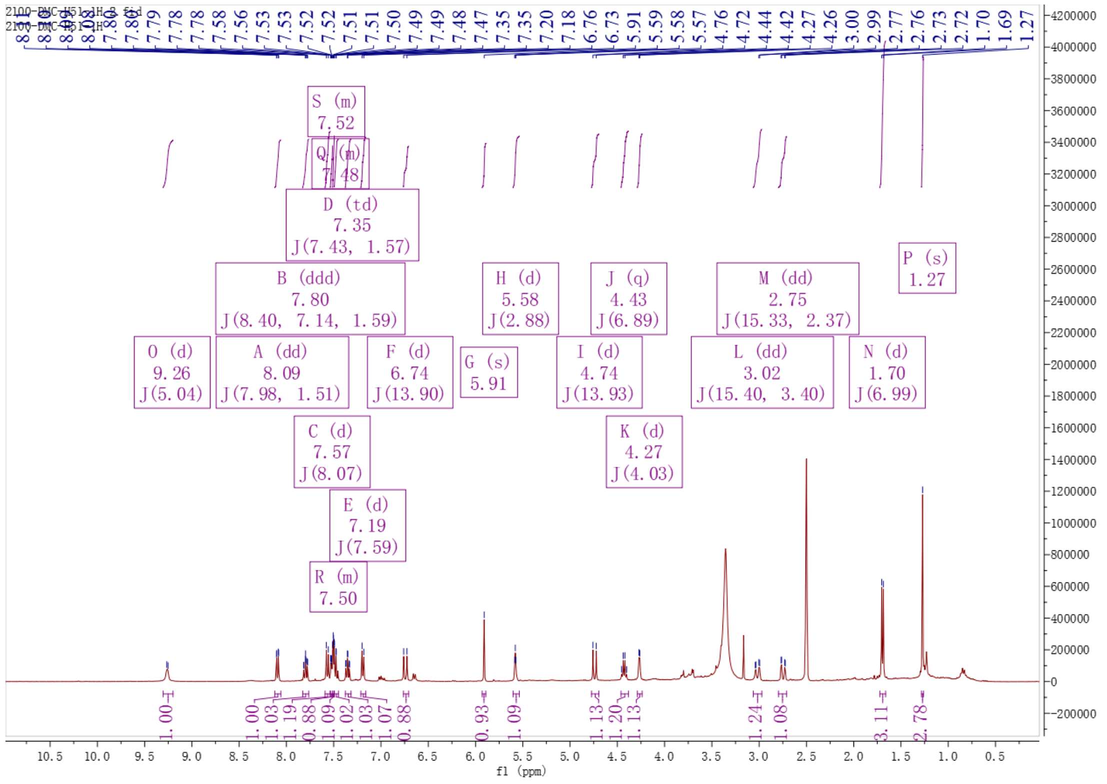

Figure S1. ^{1}H NMR (400 MHz, DMSO-d_6) spectrum of **1**

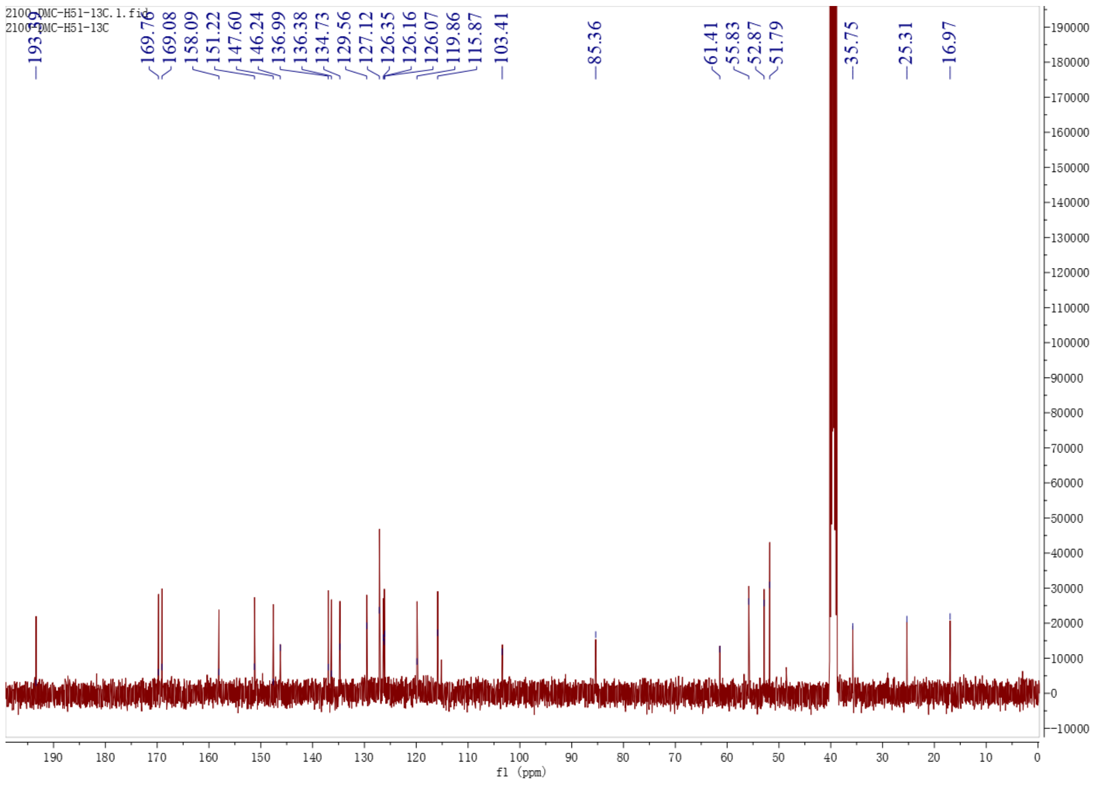

Figure S2. ^{13}C NMR (100 MHz, DMSO-d_6) spectrum of **1**

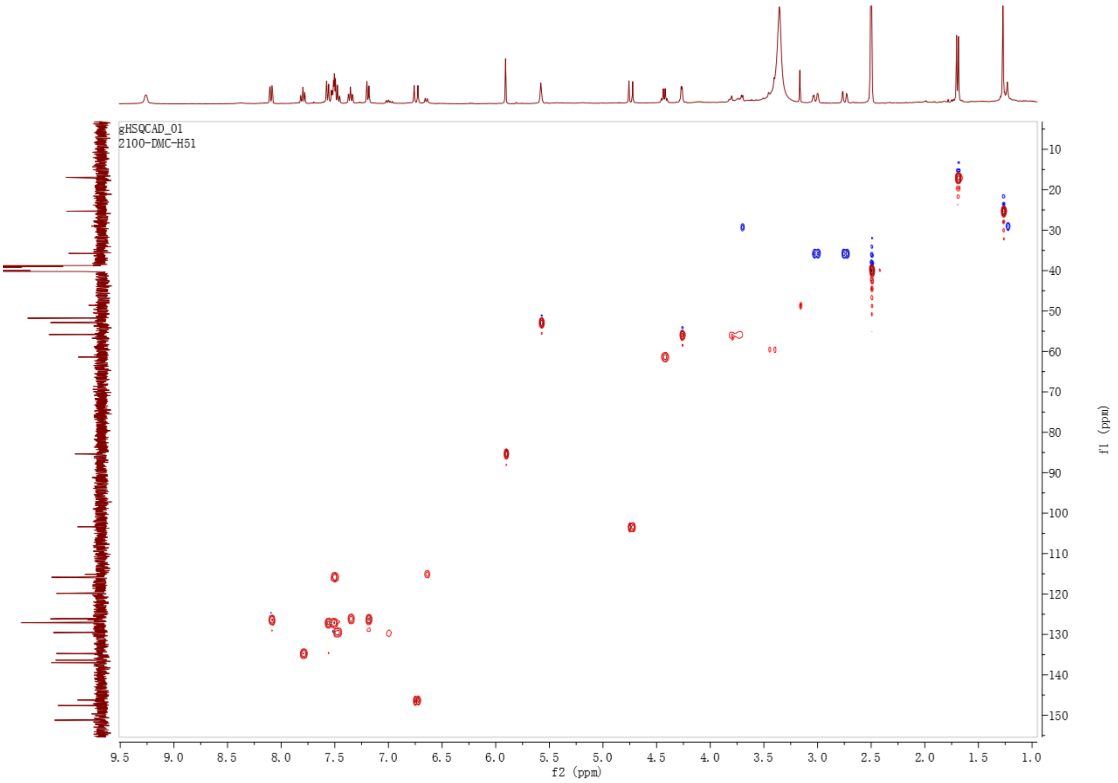

Figure S3. HSQC (DMSO-d_6) spectrum of **1**

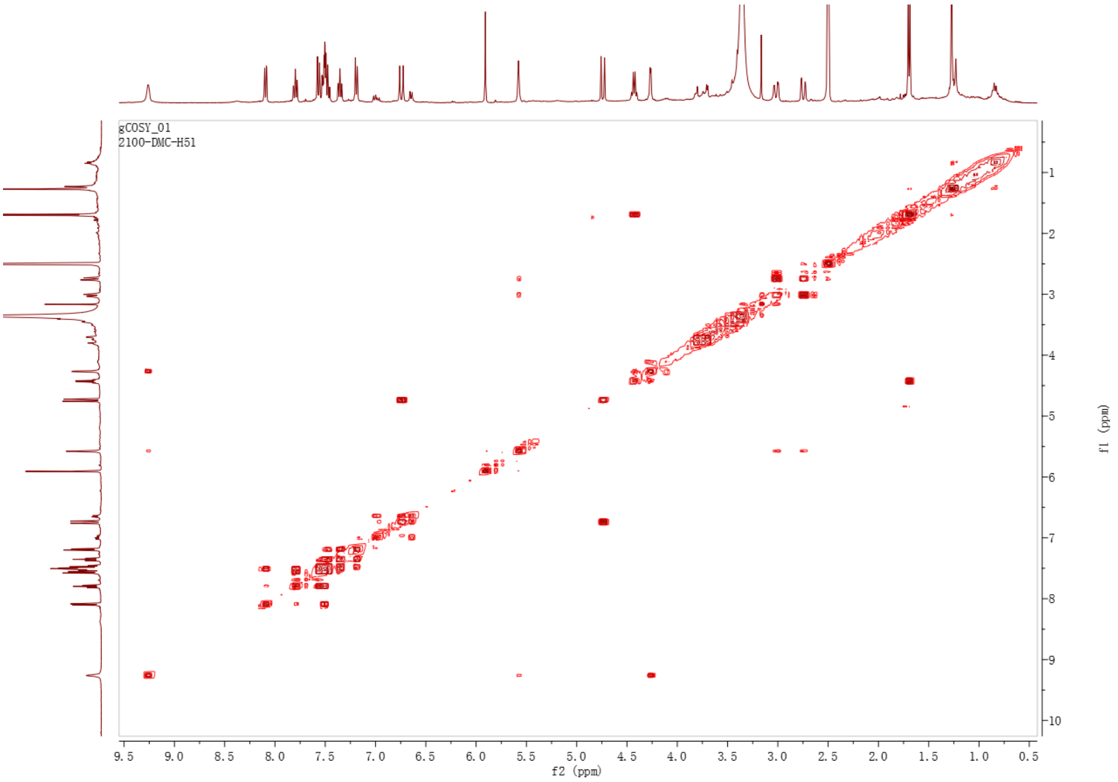

Figure S4. ^1H-^1H COSY (DMSO-d_6) spectrum of **1**

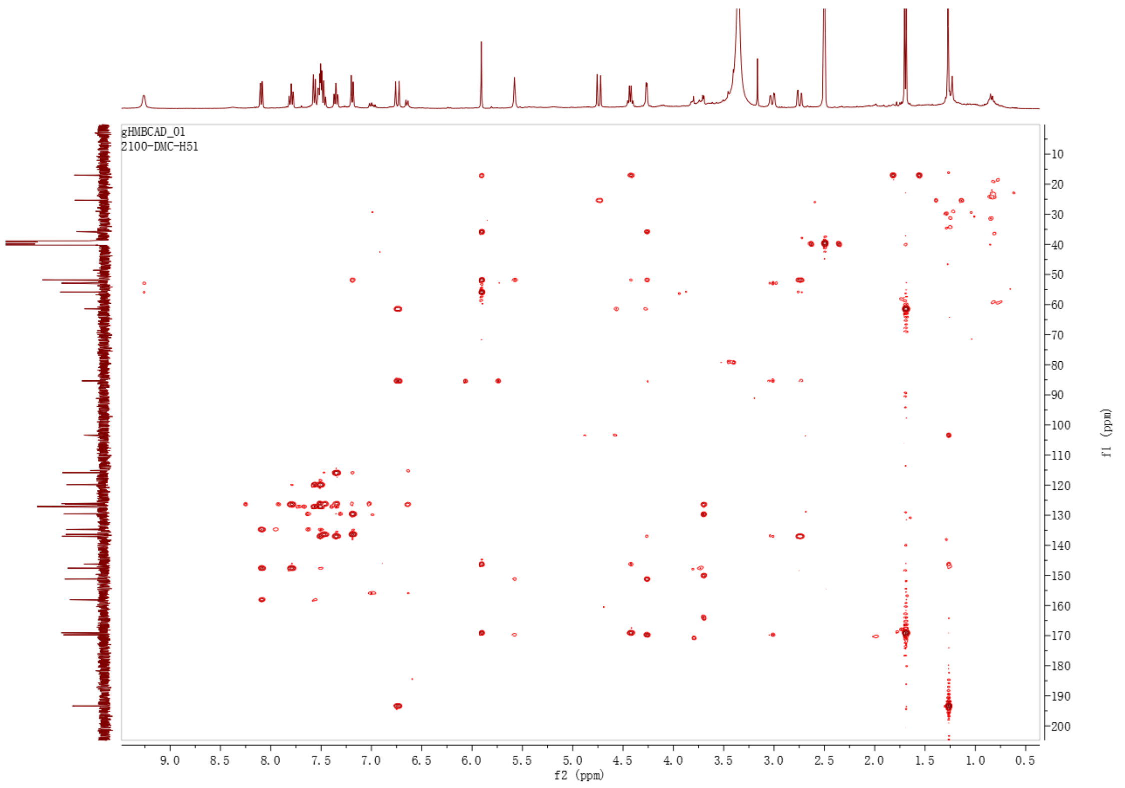

Figure S5. HMBC (DMSO-d_6) spectrum of **1**

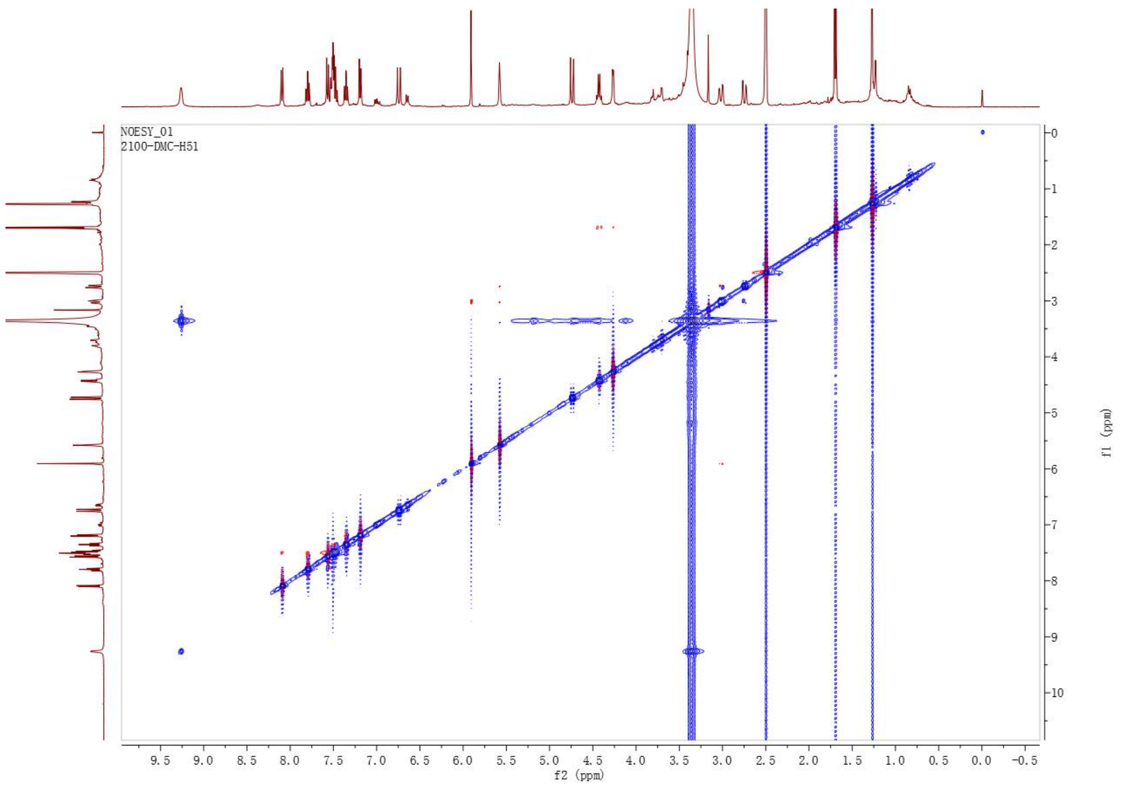

Figure S6. NOESY (DMSO-d_6) spectrum of **1**

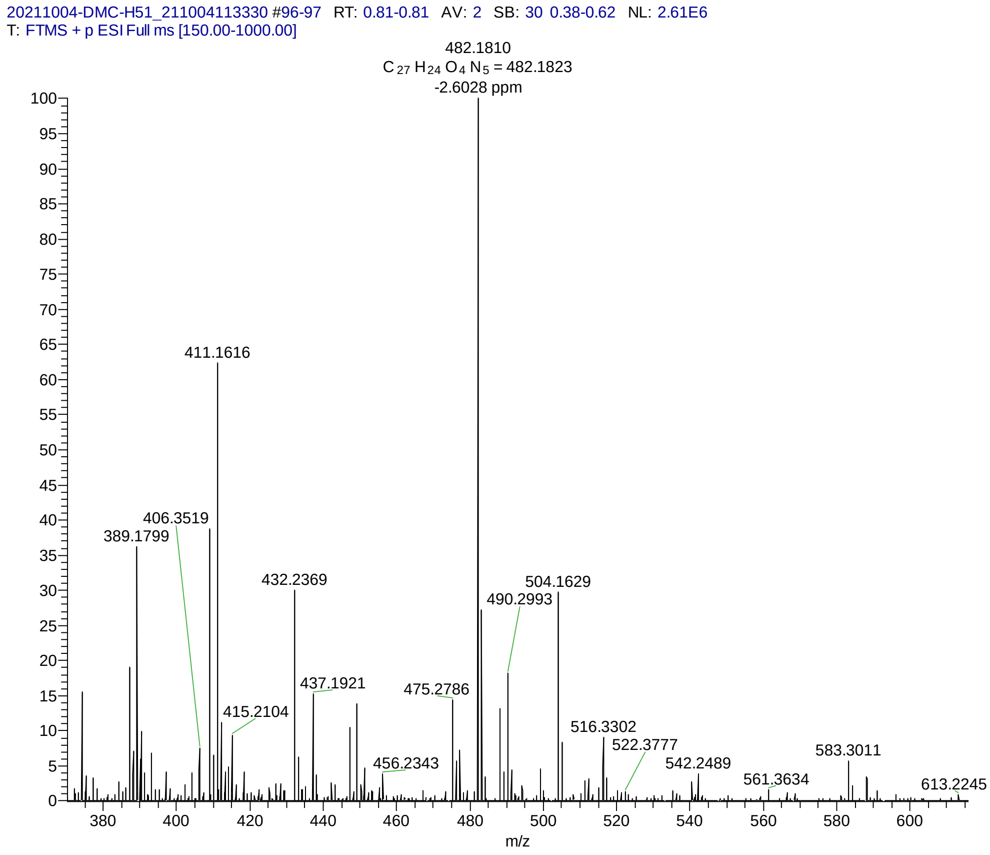

Figure S7. HRESIMS spectrum of **1**

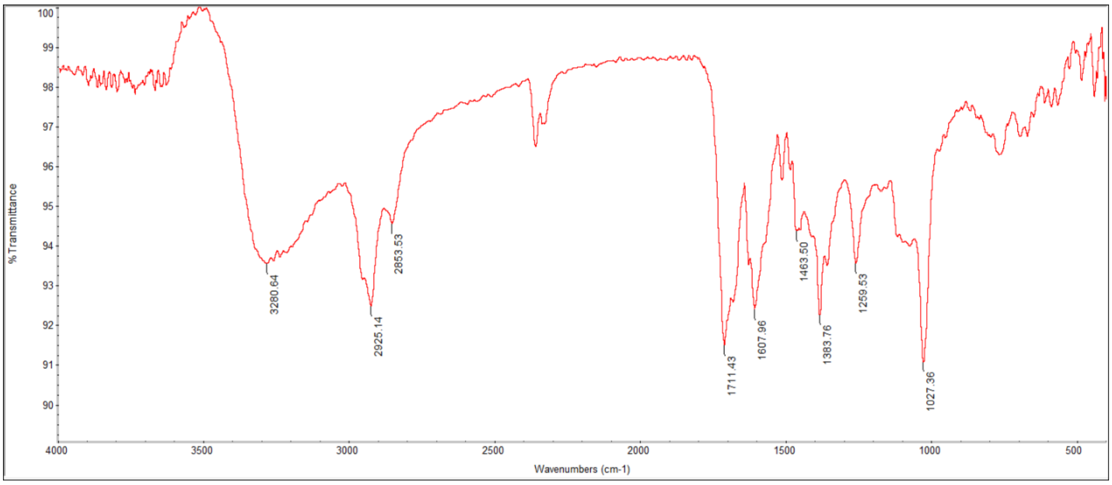

Figure S8. IR spectrum of **1**

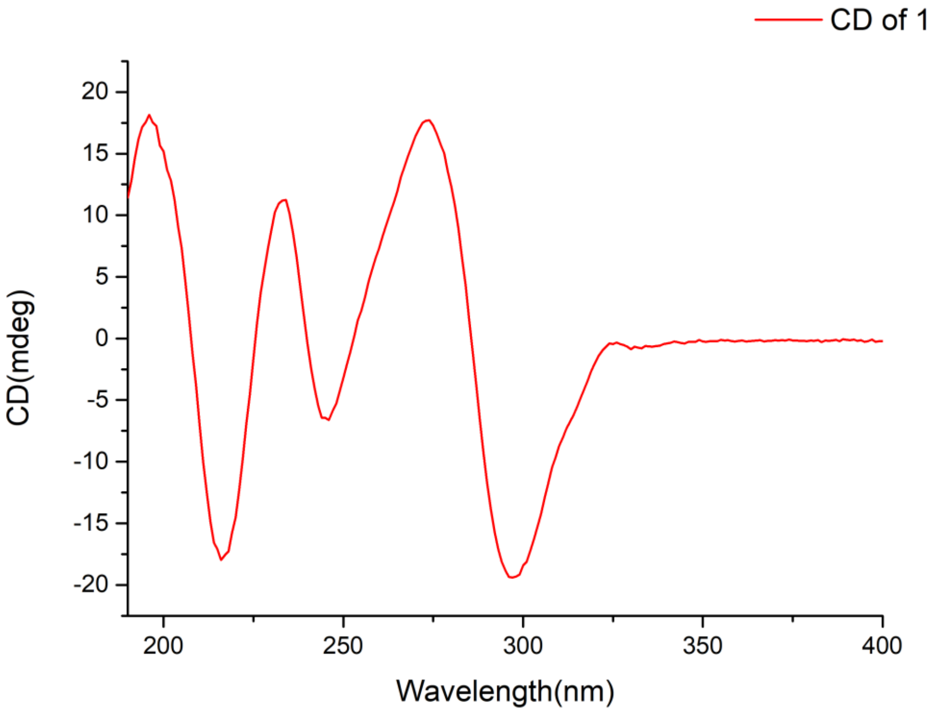

Figure S9. CD spectrum of **1**

Table S1 The antibacterial activity results (only sections are shown)

NO.	MIC (μg/L)		
	V. vulnificus	*V. rotiferianus*	*V. campbellii*
**4**	6.25	-	-
**7**	12.5	12.5	6.25
ampicillin sodium	6.25	3.12	3.12

Note: ampicillin sodium as positive control

Table S2 The anti-inflammatory activity against NO production induced by LPS (only sections are shown)

IC_{50} (μmol/L)				
**2**	**3**	**6**	**7**	Dex
66.15	37.08	37.48	61.12	13.02

Note: Dex (dexamethasone) as positive control

Table S3 The proliferation inhibitory activity against human tumor cell lines (only sections are shown)

	Inhibition ratio (%)				
	PA-1	MCF7	HCT 116	A-375	GBC-SD
**1** (10 μmol/L)	29.81	28.96	38.19	45.03	30.66
Doxorubicin (10μmol/L)	98.72	62.33	63.74	90.06	53.49

Note: doxorubicin as positive control

Table S4 Eleven culture media for small scale fermentation and their composition

Medium	Medium composition
YES	Sucrose 150 g/L, yeast extract 20 g/L, $MgSO_4 \cdot 7H_2O$ 1 g/L, $CuSO_4$ 0.01 g/L, $FeSO_4$ 0.01 g/L, $ZnSO_4$ 0.01 g/L, sea salt 15 g/L.
PDB	Potato 200 g/ L, glucose 20 g/L, sea salt 20 g/L.
GPY	Tryptone 10 g/L, yeast extract 10 g/L, glucose 20 g/L, sea salt 20 g/L.
Czapek	Sucrose 30 g/L, $NaNO_3$ 3 g/L, KH_2PO_4 1g/L, $MgSO_4 \cdot 7H_2O$ 0.5 g/L, KCl 0.5 g/L, $FeSO_4$ 0.01 g/L, sea salt 20 g/L.
Starch	Starch 10 g/L, tryptone 1 g/L, sea salt 20 g/L.
CYA	Sucrose 30 g/L, yeast extract 10 g/L, $NaNO_3$ 1 g/L, $MgSO_4$ 0.5 g/L, KCl 0.5 g/L, KH_2PO_4 1 g/L, $FeSO_4$ 0.01 g/L, sea salt 20 g/L.
TBI	Sucrose 30 g/L, KH_2PO_4 1 g/L, L-arginine 0.871 g/L, $MgSO_4$ 0.5 g/L, KCl 0.5 g/L, $FeSO_4$ 0.01 g/L, sea salt 20 g/L.
GMM	Glucose 10 g/L, 20 × salt solutions 50 mL/L, trace element 1 mL/L. 20 × salt solutions: $NaNO_3$ 120 g/L, KCl 10.4 g/L, $MgSO_4 \cdot 7H_2O$ 10.4 g/L, KH_2PO_4 30.4 g/L. trace element: $ZnSO_4 \cdot 7H_2O$ 2 g/L, H_3PO_3 1.1 g/L, $MnCl_2 \cdot 4H_2O$ 0.5 g/L, $FeSO_4 \cdot 7H_2O$ 0.5 g/L,

	$CoCl_2 \cdot 5H_2O$ 0.16 g/L, $CuSO_4 \cdot 5H_2O$ 0.16 g/L, $(NH_4)_6Mo_7O_{24}$ 0.11 g/L, Na_4EDTA 5 g/L.
MEB	Malt extract 17.0 g/L, tryptone 3.0 g/L, PH 5.4 ± 0.2, sea salt 20 g/L。
Czapek+1% tryptone	Sucrose 30 g/L, $NaNO_3$ 3 g/L, KH_2PO_4 1g/L, $MgSO_4 \cdot 7H_2O$ 0.5 g/L, KCl 0.5 g/L, $FeSO_4$ 0.01 g/L, tryptone 10 g/L, sea salt 20 g/L.
Czapek+1% yeast extract	Sucrose 30 g/L, $NaNO_3$ 3 g/L, KH_2PO_4 1g/L, $MgSO_4 \cdot 7H_2O$ 0.5 g/L, KCl 0.5 g/L, $FeSO_4$ 0.01 g/L, yeast extract 10 g/L, sea salt 20 g/L.

Table S5 Three culture media for lab scale fermentation and their composition

Medium	Medium composition (bottle)
Rice mixed GPY medium	Rice 80 g, yeast extract 0.8 g, tryptone 0.8 g, glucose 1.6 g, sea salt 4 g, water 80 mL.
Wheat mixed Czapek medium	Wheat 20 g, rice 60 g, sucrose 1.8 g, $NaNO_3$ 0.18 g, KH_2PO_4 0.06g, $MgSO_4 \cdot 7H_2O$ 0.03 g, KCl 0.03 g, $FeSO_4$ 0.0006 g, sea salt 1.2 g, water 60 mL.
GPY medium	Tryptone 2 g, yeast extract 2 g, glucose 4 g, sea salt 40 g, water 200 mL.

The rDNA-ITS sequence of the fungal strain *Metarhizium* sp. P2100

The rDNA-ITS sequence of P2100 (5′→3′):

AACCCATGTGAACTTATACCTTTTACTGTTGCTTCGGCGGGATCGCCCCGGGAAACAGGTT
CGCGAGAGCCCGGCCCCCGGAACCAAGGCGCCCGCCGGTGGGATTTACAGAACTCTTGTATGT
TTTTTTGCACGTCTGAGTGGATCATATATCAATGAATCAAAACTTTCAACAACGGATCTCTTGGT
TCTGGCATCGATGAAGAACGCAGCGAAATGCGATAAGTAATGTGAATTGCAGAATTCAGTGAA
TCATCGAATCTTTGAACGCACATTGCGCCCGCCAGTATTCTGGCGGGCATGCCTGTTCGAGCGTC
ATTTCAACCCTCAAGCCCGCAGCGGCTTGGTGTTGGGGGCCGGCAATAGTGCCTCGCGTTGTAT
GATGCGAGCGCGCGCCGCCCCCGAAATGAATTGGCGGTCTCGTCGCGGCCTCCTCTGCGTAGTA
GCATAAACCTCGCAACGGGAGCGCGGCGCGGCCACTGCCGTCAAACGCCCAACTTTTTCTCAA
GAGT
